# Climate factors drive the local adaptation of Old World cattle

**DOI:** 10.1002/imt2.70108

**Published:** 2026-02-09

**Authors:** Luyang Sun, Liangliang Jin, Ting Sun, Chenqi Bian, Fuwen Wang, Shuang Li, Hao Li, Jianyong Liu, Jicai Zhang, Johannes A. Lenstra, Artem Nedoluzhko, Zhuqing Zheng, Ruidong Xiang, Wai Yee Low, Wangdui Basang, Zhuzha Basang, Bin Li, Quji Suolang, Li Zhao, Wangmu Silang, Luobu Ciren, Zhandui Pubu, Qiangma Xire, Yanliang Song, Yuanpeng Gao, Dawei Wei, Rende Song, Min‐Sheng Peng, Bizhi Huang, Yun Ma, Xiaoting Xia, Chuzhao Lei, Ningbo Chen

**Affiliations:** ^1^ Key Laboratory of Animal Genetics, Breeding and Reproduction of Shaanxi Province, College of Animal Science and Technology Northwest A&F University Yangling China; ^2^ College of Animal Science and Technology Ningxia University Yinchuan China; ^3^ Yunnan Academy of Grassland and Animal Science Kunming China; ^4^ Faculty of Veterinary Medicine Utrecht University Utrecht The Netherlands; ^5^ Paleogenomics Laboratory European University at St. Petersburg St. Petersburg Russia; ^6^ Academy of Sciences of Sakha (Yakutia) Yakutsk Russia; ^7^ College of Food and Biology Jingchu University of Technology Jingmen Hubei China; ^8^ Faculty of Veterinary and Agricultural Science The University of Melbourne Parkville Australia; ^9^ The Davies Research Centre, School of Animal and Veterinary Sciences University of Adelaide Roseworthy Australia; ^10^ Institute of Animal Husbandry and Veterinary Science Tibet Academy of Agricultural and Animal Husbandry Sciences Lhasa China; ^11^ College of Veterinary Medicine Northwest A&F University Yangling China; ^12^ Qinghai Animal and Veterinary Sciences Work Station Yushu China; ^13^ Key Laboratory of Genetic Evolution & Animal Models and Yunnan Key Laboratory of Molecular Biology of Domestic Animals, Kunming Institute of Zoology Chinese Academy of Sciences Kunming China; ^14^ Kunming College of Life Science University of Chinese Academy of Sciences Kunming China; ^15^ Sino‐Africa Joint Research Center Chinese Academy of Sciences Kunming China

## Abstract

Climate adaptation genomics of cattle. We comparatively analyzed genomic data from 85 Old World cattle breeds/populations of Eurasia and Africa to identify genetic factors associated with climate factors. Environmental genome‐wide association studies revealed 3165 single‐nucleotide polymorphisms (SNPs) with strong correlations between climate factors and allele frequencies. We also identified loci that were significantly associated with temperature factors in the regulatory regions, such as promoter regions, of genes (*ANKRD11*, *RPL13*, *CDK10*, and *VPS9D1*) under strong selection pressure on *Bos taurus* autosome 18, which might regulate the expression of these genes. These significantly associated SNPs could be used as potential resources for marker‐assisted breeding programs aimed at climate resilience and sustainable cattle production under changing environmental conditions.

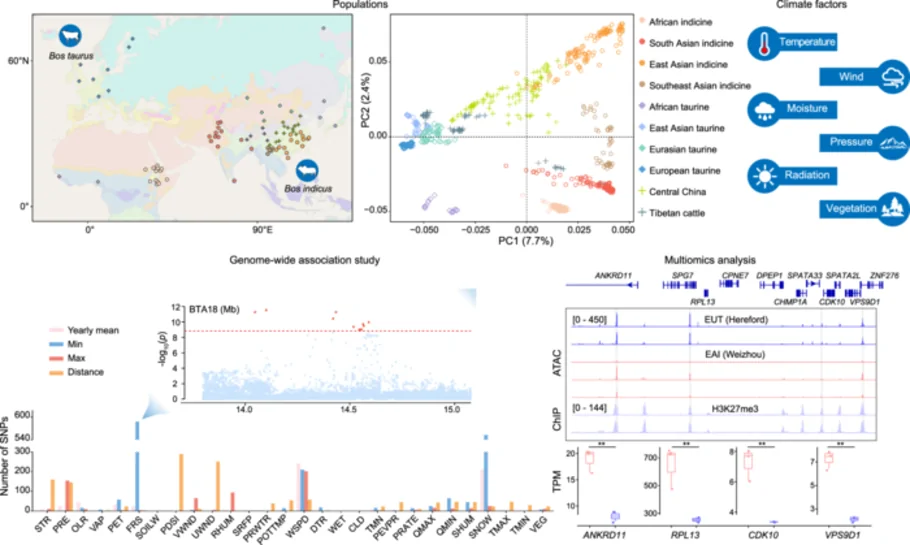

## AUTHOR CONTRIBUTIONS

**Luyang Sun**: Visualization; investigation; writing—original draft; formal analysis. **Liangliang Jin**: Investigation; writing—review and editing; visualization; formal analysis. **Ting Sun**: Writing—review and editing; visualization; investigation. **Chenqi Bian**: Visualization. **Fuwen Wang**: Visualization; validation. **Shuang Li**: Validation; visualization. **Hao Li**: Validation; visualization. **Jianyong Liu**: Project administration. **Jicai Zhang**: Project administration. **Johannes A. Lenstra**: Writing—review and editing; methodology. **Artem Nedoluzhko**: Writing—review and editing. **Zhuqing Zheng**: Writing—review and editing. **Ruidong Xiang**: Writing—review and editing. **Wai Yee Low**: Writing—review and editing. **Wangdui Basang**: Project administration. **Zhuzha Basang**: Project administration. **Bin Li**: Project administration. **Quji Suolang**: Project administration. **Li Zhao**: Project administration. **Wangmu Silang**: Project administration. **Luobu Ciren**: Project administration. **Zhandui Pubu**: Project administration. **Qiangma Xire**: Project administration. **Yanliang Song**: Visualization; validation. **Yuanpeng Gao**: Methodology. **Dawei Wei**: Methodology; writing—review and editing. **Rende Song**: Writing—review and editing. **Min‐Sheng Peng**: Writing—review and editing. **Bizhi Huang**: Resources; supervision; funding acquisition. **Yun Ma**: Resources; supervision; funding acquisition. **Xiaoting Xia**: Resources; supervision; funding acquisition; writing—review and editing. **Chuzhao Lei**: Conceptualization; funding acquisition; writing—review and editing; supervision; resources. **Ningbo Chen**: Data curation; conceptualization; writing—review and editing; supervision; resources; funding acquisition. All authors have read the final manuscript and approved it for publication.

## CONFLICT OF INTEREST STATEMENT

The authors declare no conflicts of interest.

## ETHICS STATEMENT

All protocols used in this study were approved by the Ethics Committee of Northwest A&F University (IACUC Approval No. 20240812). Blood samples and ear tissues were collected during routine veterinary treatments with the consent and logistical support of relevant agricultural institutions in each country. All procedures involving sample collection and experiments were conducted in accordance with the approved ethical guidelines.

To the Editor,

Climate change exerts major selective pressure, driving local adaptation, as evidenced by spatial genetic variation and genotype–environment interactions. Genome‐wide scans for associations with climate variables (CVs) have emerged as an effective approach to elucidate the genetic basis of this adaptation in nonmodel species [1, 2]. Domestic cattle (*Bos taurus* and *Bos indicus*) exhibit remarkably versatile climatic adaptations, with taurine cattle exhibiting cold‐tolerant traits (large size and thick hair) and indicine cattle exhibiting heat‐ and disease‐resistance traits [3]. Crossing taurine and indicine cattle has resulted in numerous cattle breeds/populations adapted to different local environments [4, 5]. While previous genomic studies of cattle have identified candidate genes through association signals, their focus on highly productive breeds or specific geographic regions [6, 7] has limited our understanding of global climate adaptation and the underlying regulatory mechanisms, particularly those involving noncoding single‐nucleotide polymorphisms (SNPs)—due to the absence of integrated multi‐omics data. We focused on cattle populations from the Old World (Eurasia and Africa), which have undergone long‐term adaptation to diverse environmental conditions.

A wide panel of 744 cattle from 85 breeds/populations (Tables S1, 2) was divided into 10 groups: African indicine (AFI, *n* = 107), African taurine (AFT, *n* = 17), European taurine (EUT, *n* = 72), East Asian taurine (EAT, *n* = 47), Tibetan cattle (TB, *n* = 64), Eurasian taurine (EUAT, *n* = 39), South Asian indicine (SAI, *n* = 124), Southeast Asian indicine (SEAI, *n* = 41), Central China (CC, *n* = 99), and East Asian indicine (EAI, *n* = 134) (Figure 1A). After variant calling, 74,041,479 autosomal SNPs were identified. Genome‐wide nucleotide diversity is higher in indicine than in taurine cattle (Figure S1), presumably due to intense commercial breeding in taurine lineages [3]. The pairwise fixation index (*F*_ST_) reaches values of 0.502 between taurine and indicine breeds (Figure S2 and Table S3). Admixture analysis, principal component analysis (PCA), and a phylogenetic network showed clear separation of eight groups with a mixed ancestry of TB and CC (Figure 1B–E, Figure S3, and Figure S4A). PCA confirmed a phylogeographical differentiation, highlighting the contrasts between indicine and taurine cattle (PC1), between SAI and EAI populations (PC2, Figure 1C), and between EUT and Asian taurine populations (EAT and TB; PC3; Figure 1D). The population analyses also revealed crossbreeding with taurine cattle in the EAI and SAI groups. Removal of crossbred individuals resulted in a small but significant decrease in nucleotide diversity (Figure S4B). A NeighborNet graph based on *F*_ST_ values was established to provide a detailed visualization of the relationships among cattle breeds across Eurasia and Africa (Figure 1E, Figure S5, and Table S4).

**Figure 1:**
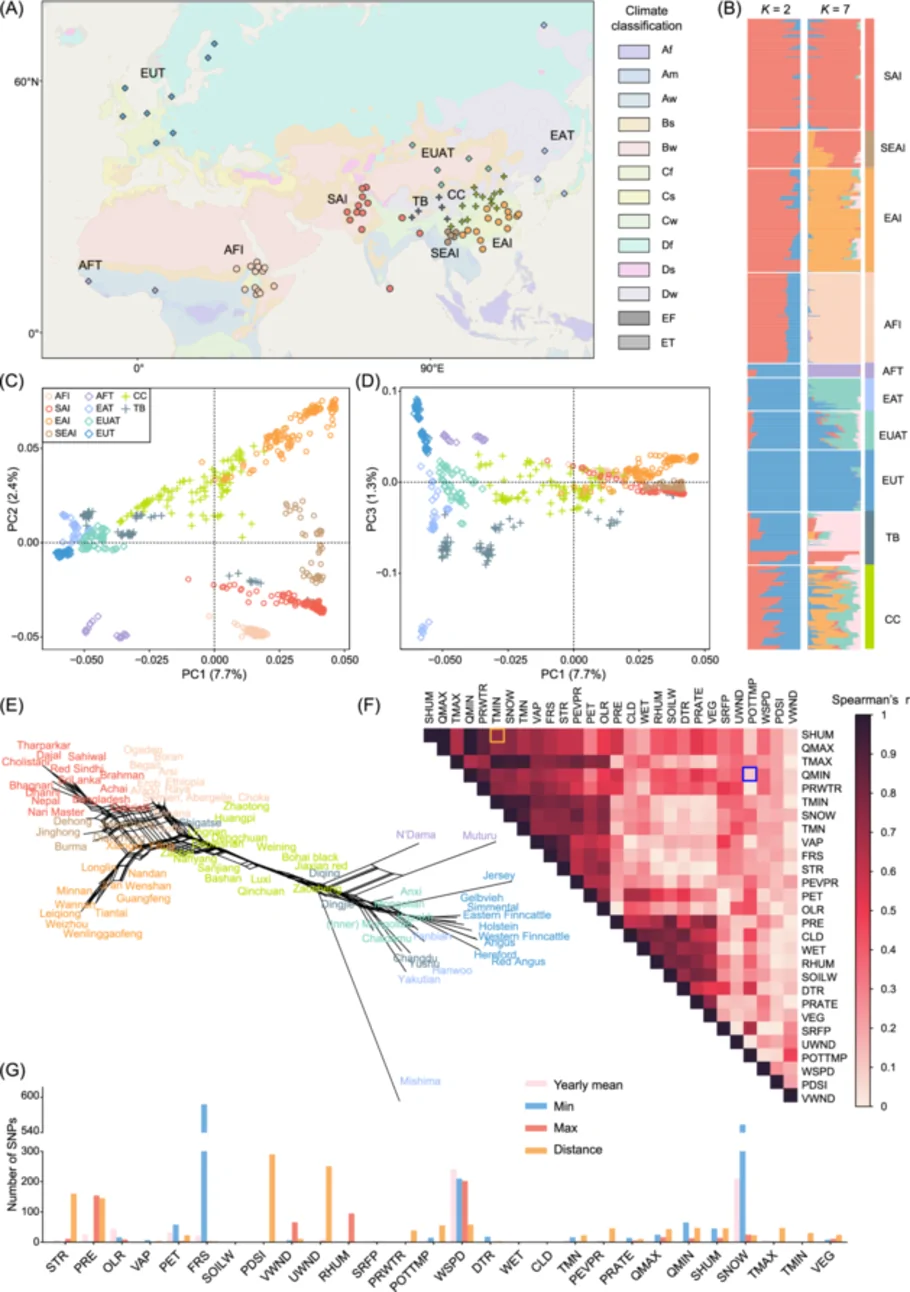
Population genomic map of cattle, environmental variables, and candidate SNPs. (A) Geographic distribution of cattle breeds/populations worldwide. Sampling locations of the 85 breeds/populations shown are: African indicine (AFI), African taurine (AFT), European taurine (EUT), East Asian taurine (EAT), Tibetan cattle (TB), Eurasian taurine (EUAT), South Asian indicine (SAI), Southeast Asian indicine (SEAI), Central China (CC), and East Asian indicine (EAI). The Köppen–Geiger climate classification map was generated by the National Earth System Science Data Center, National Science & Technology Infrastructure of China (http://www.geodata.cn): A, tropical; f, rainforest; m, monsoon; w, savannah. B, arid; w, desert; s, steppe. C, temperate; s, dry summer; w, dry winter; f, without a dry season. D, cold; s, dry summer; w, dry winter; f, without a dry season. E, polar; T, tundra; F, frost. (B) Admixture analysis of cattle populations showing ancestry proportions at *K* = 2 and *K* = 7 (optimal *K*, CV error = 0.245). (C) Principal component analysis (PCA) of all 744 cattle, illustrated by PC1 versus PC2 and (D) PC1 versus PC3. The colors reflect the geographic regions of the samples. (E) Phylogenetic network showing the relationships among the breeds identified using SplitsTree4. (F) Heatmap showing the absolute values of Spearman's rank correlation coefficients for the annual mean values of the 28 climate factors. The 28 environmental variables and their abbreviations are as follows: surface temperature (STR), outgoing longwave radiation (OLR), potential temperature (POTTMP), air temperature (TMN), maximum temperature (TMAX), minimum temperature (TMIN), palmer drought severity index (PDSI), maximum specific humidity (QMAX), specific humidity (SHUM), minimum specific humidity (QMIN), relative humidity (RHUM), precipitable water (PRWTR), precipitation rate (PRATE), potential evaporation rate (PEVPR), wind speed (WSPD), U wind component (UWND), surface pressure (SRFP), vapor pressure (VAP), potential evapotranspiration (PET), ground frost frequency (FRS), soil moisture (SOILW), V wind component (VWND), diurnal temperature range (DTR), wet day frequency (WET), cloud cover (CLD), snow cover (SNOW), total precipitation (PRE), and vegetation cover (VEG). Specific correlations discussed in the text are highlighted: a strong correlation (TMIN vs. SHUM) is marked in orange, and a nonsignificant correlation (QMIN vs. POTTMP) is marked in blue. (G) Comparative distribution of candidate SNPs among different environmental variables. For each of the 28 variables, four bars indicate the number of SNPs significantly associated with its Yearly mean, Min (minimum), Max (maximum), and Distance (range, Max–Min).

Environmental variables exhibited complex interrelationships (Tables S5, 6). Their monthly distributions revealed distinct climatic patterns among the cattle populations (Figures S6–S10). PCA of these variables revealed significant differences between the cattle populations (Figure S11). Geographically close groups generally experience similar environments, with notable exceptions, for example, SAI breeds, which are geographically close but occupy a large range of environmental niches [3]. Conversely, Dianzhong (Yunnan, China) and Abergelle (Africa) cattle occupy similar environmental niches despite their geographic distance. A strong correlation (Figure 1F) between minimum temperature (TMIN) and specific humidity (SHUM), but not between minimum specific humidity (QMIN) and potential temperature (POTTMP), highlights the complex interrelationships among climatic factors.

We integrated three methods (GEMMA, LFMM, and BayPass) for a robust genome‐wide association study (GWAS) to assess the correlation between the allele frequency of each SNP and environmental CVs (Figures S12, 13). This yielded 3165 high‐confidence SNPs associated with 28 CVs (Table S7), predominantly in introns (50.8%) with a twofold enrichment of exonic SNPs (1.4%; Figure S13B). Among all CVs, the number of SNPs associated with snow cover (SNOW) and ground frost frequency (FRS) was the highest, totaling 1269 (40.1%); in contrast, wet day frequency (WET) and cloud cover (CLD) each exhibited only one significantly associated SNP (WET: *p* = 6.8E^−10^, Bayes factor (BF) = 33.9; CLD: *p* = 2.2E^−11^, BF = 32.2; Figure 1G). Results of functional enrichment analysis revealed transcriptional regulation, bacterial response, axon guidance, glutamatergic synapses, and cAMP/Rap1 signaling as factors linked to environmental adaptation (Figure S13C, Table S8).

Temperature is the key meteorological variable that directly affects the thermal comfort of cattle. A GWAS of the associations between SNPs and eight temperature‐related CVs (for full definitions, see Table S5) identified 1584 significantly associated SNPs. A total of 81 top‐ranking loci associated with temperature were identified, including breed‐specific SNPs in Yakutian cattle from the Arctic Circle and Tibetan cattle from the highland region. In Yakutian cattle, we detected 70 breed‐specific SNPs associated with potential temperature (POTTMP), SNOW, and surface temperature (STR) in 30 genes (Figure 2A and Figure S14), including *ABLIM1* and *TCF7L2*. *ABLIM1* regulates muscle development [8], and *TCF7L2* is a key regulator of adipocyte metabolism and adaptive fat deposition [9], where it functions within a PPARγ/lncRNA axis to restrain thermogenesis and maintain adipose plasticity [10]. In Tibetan cattle, we detected 11 breed‐specific SNPs associated with SNOW, FRS, POTTMP, and STR, including variants in *DUOX1*—a gene essential for thyroid hormone synthesis, which was under strong natural selection in Tibetan cattle [6]. We also identified novel associated SNPs in *PTK2* and *PDE10A*, both of which are involved in vascular morphogenesis. *PTK2* has been identified to be under selective pressure in both Tibetan sheep [11] and chickens [12], indicating a fundamental role in high‐altitude adaptation across species.

**Figure 2:**
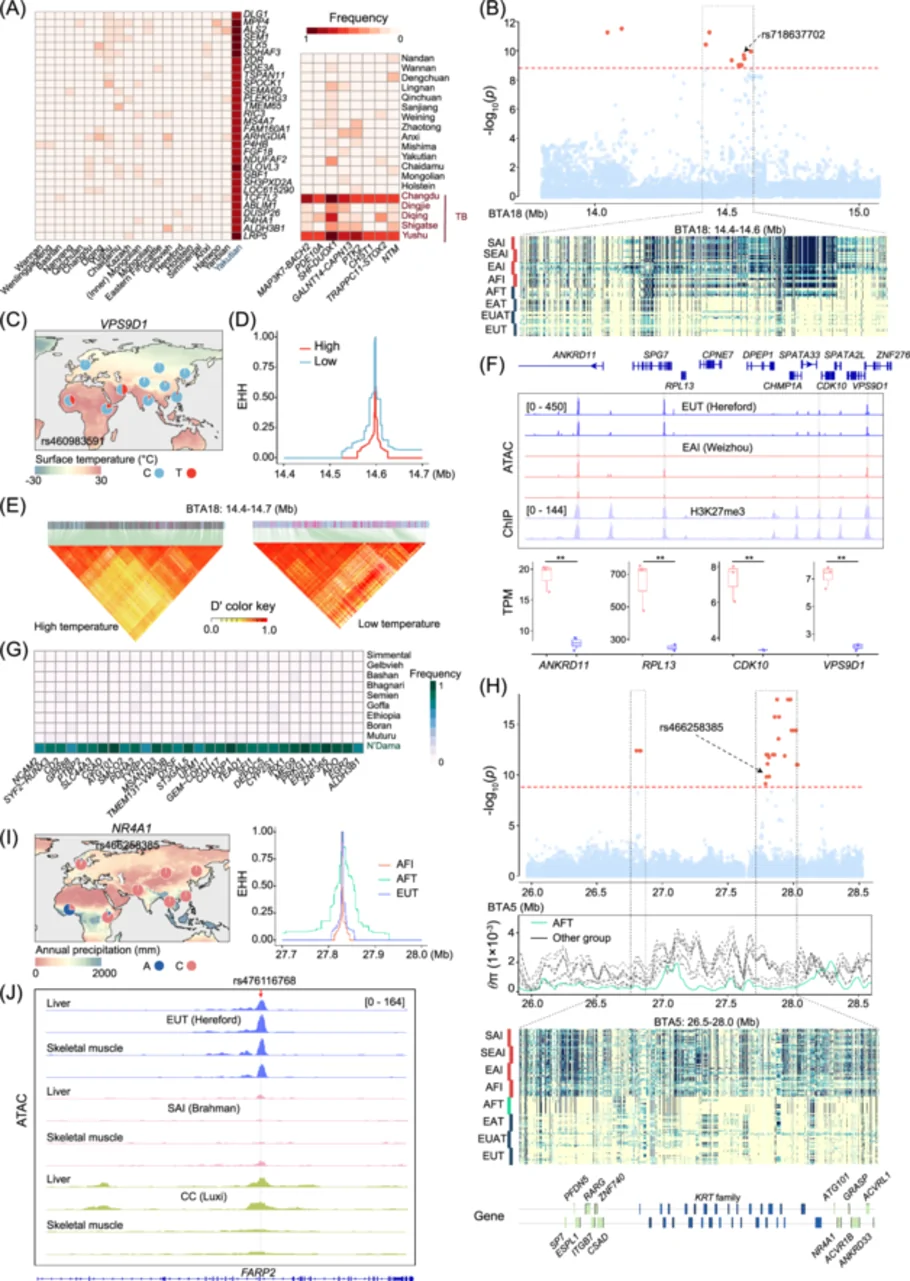
Adaptation‐related regions within genes. (A) Frequencies of group‐specific substitutions in adaptive genes in Yakutian (blue) and Tibetan (red) cattle. (B) Associations of genetic variants with FRS on *Bos taurus* autosome (BTA) 18 based on the LMM models, and haplotype structures for the candidate regions. The red dashed line indicates the genome‐wide significance threshold. (C) Geographical distributions of the allele frequency of rs460983591 in global cattle groups. The colors on the map represent the variations in surface temperature across different regions. The reference locus is blue, and the derived loci is red. (D) Decay of extended haplotype homozygosity (EHH) around rs472613982 in groups of cattle that survived at different ambient temperatures. The lower temperature groups include EAT, EUAT, and EUT cattle. The higher temperature groups include SAI, SEAI, EAI, AFI, and AFT cattle. (E) Linkage disequilibrium block of the groups of cattle living at different ambient temperatures. (F) The chart, created with the Integrated Genomics Viewer (IGV), shows the regulatory effects of candidate functional variants on BTA18 (14.4–14.6 Mb). Gene expression levels (TPM, Transcripts Per Million) in Hereford (EUT, cold‐adapted, blue; *n* = 2) and Weizhou (EAI, heat‐adapted, red; *n* = 3) cattle skeletal muscle obtained from RNA‐seq are shown. Significance was determined using the empirical Bayes moderated *t*‐test (**p < 0.01). (G) Frequencies of lineage‐specific substitutions in adaptive genes of N'Dama cattle. (H) Associations of genetic variants with PRE on BTA5 based on the LMM model. Selective sweep signals based on the *θ*π of the cattle groups are plotted. Haplotype structures for significant regions. The red dashed line indicates the genome‐wide significance threshold. The other groups include EAT, EUAT, EUT, SAI, SEAI, EAI, and AFI cattle. Gene annotations in the sweep regions are indicated at the bottom. Geographical distributions of the allele frequencies of rs466258385 in *NR4A1* across global cattle groups. (I) Decay of EHH around rs466258385 in AFT, AFI, and EUT. The colors on the map represent the variations in precipitation across different regions. The reference locus is shown in orange, and the mutant loci are shown in blue. (J) Chromatin accessibility at the rs476116768 locus in the *FARP2* gene, profiled by ATAC‐seq in skeletal muscle and liver of Hereford (EUT, blue; *n* = 2), Brahman (SAI, pink; *n* = 2), and Luxi (CC, green; *n* = 2) cattle.

Under similar environmental pressures, geographically separated populations can converge on analogous beneficial alleles. Our multi‐population selection scan revealed a signature of such convergent evolution. There are 43 interesting SNPs associated with FRS and SNOW (*p* < 1.4E^−10^, BF > 30), with strong signals in the ~200 kb region for *B. taurus* autosome (BTA) 18 (14.4–14.6 Mb) in EAT, EUT, and EUAT cattle, but not in AFT cattle (Figure 2B and Figure S15A). The haplotype structure diverges between cattle from hot and cold climates (Figure 2B). SNP rs460983591 showed the highest allele frequency divergence between groups (Figure 2C). Further evidence for recent selection originated from the slower linkage disequilibrium (LD) decay in cold‐adapted populations (Figure S15B) and significantly slower extended haplotype homozygosity (EHH) decay for the missense mutation rs472613982 (Figure 2D). Cold‐adapted cattle presented multiple LD blocks with predominant ancestral haplotypes, whereas the heat‐adapted cattle demonstrated abundant derived haplotypes (Figure 2E), suggesting that selective pressure maintains ancestral haplotypes in cattle in cold environments, while thermal heterogeneity favors diverse derived haplotypes in hot climates. This region included genes associated with epidermal growth factor signaling (*CHMP1A*), thermal sensitivity (*CDK10*) [13], lipid peroxidation (*DPEP1* and *CHMP1A*) [14], and ribosome function (*RPL13*). To explore the potential regulatory roles of associated SNPs in this region, we integrated key tissues from EUT (Hereford) cattle and EAI (Weizhou) cattle for RNA sequencing and assay for transposase‐accessible chromatin with sequencing (ATAC‐seq). We identified different peaks at the promoters of *ANKRD11*, *RPL13*, *CDK10*, and *VPS9D1* in EUT and EAI cattle (FDR < 0.01; Figure 2F; Tables S9, 10). In contrast, RNA‐seq showed decreased expression of these genes (*p* < 0.05), while chromatin immunoprecipitation sequencing showed H3K27me3 enrichment. Notably, high *RPL13* expression promotes the repair of ultraviolet damage [15], and *CDK10* is thermally sensitive [13]. Our results indicate that the significantly associated and selected SNPs located in non‐coding regions may regulate gene expression by altering regulatory elements, thereby exerting their gene function.

Moisture critically shapes cattle respiratory efficiency, skin barrier function, and pathogen exposure risk. A GWAS of the associations between SNPs and 13 moisture‐related CVs (for full definitions, see Table S5) identified 870 significantly associated SNPs. We identified 115 candidate SNPs specific to N'Dama cattle that are associated with total precipitation (PRE), potential evaporation rate (PEVPR), and precipitation rate (PRATE) (Figure 2G and Figure S16). These SNP‐annotated genes are clearly variety‐specific and may be associated with distinctive adaptations. Among these, 23 SNPs are located near the *CDH17* gene, which is believed to be expressed in intestinal epithelial cells and is related to kidney development. The missense mutation rs111440168 in *CDH17* exhibited a linked haplotype and a high EHH value in N'Dama cattle (Figure S17). Three candidate SNPs within the *SLAMF1* gene were identified for BTA3 and were associated with PRATE. The two missense mutations in *SLAMF1* (rs110202396: LFMM, *p* = 4.63E^−12^; LMM, *p* = 5.96E^−10^; rs108975404: LFMM, *p* = 8.19E^−12^; LMM, *p* = 6.97E^−10^) are present only in taurine breeds, and their allele frequencies are significantly higher in regions with high precipitation (Figure S18), except in TB cattle. Three SNPs introducing mutations in *ARFGEF3* (also known as *BIG3*) occurred in indicine cattle (Figure S19). This gene encodes an Arf‐GTP exchange factor that negatively regulates insulin granule biogenesis and secretion through its localization in the trans‐Golgi network [16]. *ARFGEF3* deficiency leads to accelerated proinsulin processing [16], increased insulin secretion, and impaired glucose homeostasis. Our multi‐population selection scan revealed a convergent adaptation signature on BTA5 (26.8–27.8 Mb). We identified 10 nonoverlapping SNPs associated with PRE (*p* < 6.36E^−10^, BF > 57) that were under selection in AFT cattle (Figure 2H), containing genes involved in alveolar development and immune regulation. A nucleotide diversity analysis gave a strong selective signal and a distinct fixed haplotype in AFT cattle (Figure 2H). A missense mutation in *NR4A1* (rs466258385) yielded a high derived allele frequency of 0.91 in AFT cattle (Figure 2I). Moreover, haplotypes carrying the derived allele (G) exhibited a higher EHH than the haplotypes carrying the ancestral allele (T). The association of this locus with precipitation‐related variables was coupled with the known functions of the encompassing genes in lung function (*ACVRL1* and *ACVR1B*) [17] and immune regulation (*NR4A1* and *ATG101*) [18]. However, further functional validation is required to establish a direct causal relationship.

With 98.6% of the identified SNPs localized in noncoding regions (Table S7), we further investigated whether our GWAS hits might serve as regulatory variants by examining their overlap with established cattle cis‐expression quantitative trait loci (cis‐eQTL) [19]. This analysis aimed to elucidate the functional regulatory logic of the noncoding SNPs. We identified 208 potential eQTLs (*p* < 0.05) overlapping with our associated SNPs (Table S11). Overall, 15.36% (208/3165) of the associated SNPs overlapped with eQTLs. Notably, the 3'untranslated region (UTR) was significantly enriched for such relationships (42%, 6 out of 14; *p* = 0.0001, Fisher's exact test), suggesting that these mutations are preferentially enriched in cis‐regulatory elements, particularly within the 3'UTR. These associated SNPs were identified in several important candidate genes, such as *SLAMF1*, *PTK2*, and *ARFGEF3*. A region associated with the Palmer drought severity index (PDSI) was significantly colocalized (rcp = 0.96) with cis‐eQTLs for *FARP2* in the pituitary, with SNPs rs382549743 (*p* = 3.9E^−06^) and rs476116768 (*p* = 4.8E^−06^) as the top hits (Figure S20A). ATAC‐seq analysis revealed obvious peaks near rs476116768 in EUT (Hereford) cattle (Figure 2J). Moreover, ChIP‐seq revealed that this SNP occurs in the insulator region and is enriched in the TFAP2A motif (*p* = 8.7E^−04^) (Figure 2J and Figure S21). The transcription factor AP‐2α (tfap2a) is associated with nephron differentiation during kidney development [20]. The derived allele of rs476116768 was predominantly found in EUT cattle (Figure S20B), and its frequency exhibited a significant positive correlation with the range of drought fluctuation (PDSI–Distance; *R*² = 0.265, *p* = 6.3E^−07^). These results suggest that rs476116768 may participate in physiological processes related to PDSI by modulating the expression of *FARP2*. The molecular and physiological mechanisms of this adaptation remain a key subject for future investigation.

Cattle have successfully adapted to a wide spectrum of environmental conditions, spanning diverse and complex climate types. Using an integrated framework combining GWAS with climatic variables, epigenomic profiling, and cis‑eQTL mapping, we systematically identified SNPs associated with key environmental CVs, many of which were breed‐specific. Our analysis further elucidated the distinct functional contributions of coding versus non‑coding variants to environmental adaptation. Furthermore, it revealed signatures of convergent evolution, such as the repeated selection of the *PTK2* gene in distinct high‑altitude species. Candidate variants (e.g., *CDK10* for cold tolerance, *FARP2* for drought resilience) offer targets for breeding climate‐adapted cattle. We therefore conclude that our integrative framework provides an effective approach for identifying loci associated with environmental adaptation in cattle.

## Supporting information


**Figure S1.** The genetic diversity of the Old World of cattle.
**Figure S2.** Pairwise genetic differentiation (*F*_ST_) among cattle breeds/populations.
**Figure S3.** Population structure inferred from ADMIXTURE analysis.
**Figure S4.** Population genetic results.
**Figure S5.** *F*_ST_ among different cattle breeds/groups after individual selection.
**Figure S6.** Monthly density distributions of climate variables: STR, OLR, POTTMP, TMN, TMAX, and TMIN.
**Figure S7.** Monthly density distributions of climate variables: PDSI, QMAX, SHUM, QMIN, RHUM, and PRWTR.
**Figure S8.** Monthly density distributions of climate variables: PRATE, PEVPR, WSPD, UWND, SRFP, and VAP.
**Figure S9.** Monthly density distributions of climate variables: PET, FRS, SOILW, VWND, DTR, and WET.
**Figure S10.** Monthly density distributions of climate variables: CLD, SNOW, PRE, and VEG.
**Figure S11.** Principal component analysis of environmental variables across cattle breeds/populations.
**Figure S12.** Results of the core model from BayPass analysis.
**Figure S13.** Part of the results from the genome‐wide environmental association analysis.
**Figure S14.** Heatmap of lineage‐specific allele frequencies for environmental adaptation SNPs in Yakutian and Tibetan cattle.
**Figure S15.** Analysis of selection signals and linkage disequilibrium decay in temperature‐adapted cattle groups.
**Figure S16.** Heatmap of lineage‐specific allele frequencies for environmental adaptation SNPs in N'Dama cattle.
**Figure S17.** Extended haplotype homozygosity (EHH) decay and haplotype bifurcation around rs111440168.
**Figure S18.** Significant genomic region association with temperature of *SLAMF1* and *CD84* gene.
**Figure S19.** Allele frequency geographical distribution of the intron mutations rs210877007, rs382149931, and rs110707290 on the *ARFGEF3* gene.
**Figure S20.** Functional and population genetics analysis of the *FARP2* gene locus on BTA3.
**Figure S21.** Tissue‑specific chromatin profiles at the *FARP2* insulator variant rs476116768 visualized.

**Table S1.** Summary information of 85 cattle breeds/groups genotyped and sequenced.
**Table S2.** Overview of the 77 new sampling information and sequencing statistics.
**Table S3.** *F*_ST_ Matrix of 744 individuals from 85 domestic cattle breeds/populations.
**Table S4.** *F*_ST_ Matrix of 731 individuals from 85 domestic cattle breeds/populations.
**Table S5.** Summary of 28 climate variables gathered for this analysis and sources of information.
**Table S6.** Summary information of the 85 domestic cattle breeds/populations and the environmental parameters for their central range areas.
**Table S7.** Candidate SNPs identified by genome‐wide environmental association analysis.
**Table S8.** Overrepresented GO and KEGG terms among the candidate genes identified to be under environmental adaptation.
**Table S9.** Summary of ATAC‐seq, ChIP‐seq, and RNA‐seq data used in this study.
**Table S10.** Details on QC procedures for unpublished data.
**Table S11.** Association between Significant SNPs and cis‐eQTLs for *P*‐value.

## Data Availability

All the sequencing data have been deposited in the NCBI Sequence Read Archive (SRA) under accession numbers PRJNA905718 (https://www.ncbi.nlm.nih.gov/bioproject/PRJNA905718), PRJNA1085861 (https://www.ncbi.nlm.nih.gov/bioproject/PRJNA1085861), and PRJNA658727 (https://www.ncbi.nlm.nih.gov/bioproject/PRJNA658727). The data and scripts used are saved in GitHub (https://github.com/Luyang-Sun/Climate-adaptation-in-cattle.git). Supplementary materials (methods, figures, tables, graphical abstract, slides, videos, Chinese translated version, and update materials) may be found in the online DOI or iMeta Science http://www.imeta.science/. Other data that support the findings of this study are available in the Supplementary Material of this article. The data that support the findings of this study are openly available in the NCBI SRA database at https://www.ncbi.nlm.nih.gov/sra, reference numbers PRJNA1085861, PRJNA658727, and PRJNA905718.
