# Supplementary material for: Climate factors drive the local adaptation of Old World cattle
Source: Imeta. 2026 Feb 9;5(1):e70108. doi: 10.1002/imt2.70108 (PMC13726580; doi:10.1002/imt2.70108)
Supplement: Supplementary file 1 [file IMT2-5-e70108-s002.docx]

Supporting information to

*Climate Factors Drive the Local Adaptation of Old World Cattle*

Luyang Sun ^1, 2#^, Liangliang Jin ^1#^, Ting Sun^1#^, Chenqi Bian ^1^, Fuwen Wang ^1^, Shuang Li ^1^, Hao Li ^1^, Jianyong Liu ^3^, Jicai Zhang ^3^, Johannes A. Lenstra ^4^, Artem Nedoluzhko ^5, 6^, Zhuqing Zheng ^7^, Ruidong Xiang ^8^, Wai Yee Low ^9^, Wangdui Basang ^10^, Zhuzha Basang ^10^, Bin Li ^10^, Quji Suolang ^10^, Zhao Li ^10^, Wangmu Silang ^10^, Luobu Ciren ^10^, Zhandui Pubu ^10^, Qiangma Xire ^10^, Yanliang Song ^11^, Yuanpeng Gao ^11^, Dawei Wei ^2^, Rende Song ^12^, Min-Sheng Peng ^13, 14, 15^, Bizhi Huang ^3^ , Yun Ma ^2^*, Xiaoting Xia ^1^*, Chuzhao Lei ^1^*, Ningbo Chen ^1^*

^1^ Key Laboratory of Animal Genetics, Breeding and Reproduction of Shaanxi Province, College of Animal Science and Technology, Northwest A&F University, Yangling 712100, China

^2^ College of Animal Science and Technology, Ningxia University, Yinchuan 750021, China

^3^ Yunnan Academy of Grassland and Animal Science, Kunming 650212, China

^4^ Faculty of Veterinary Medicine, Utrecht University, Utrecht 3584 CL, The Netherlands

^5^ Paleogenomics Laboratory, European University at St. Petersburg, 6/1A Gagarinskaya Street, St. Petersburg 191187, Russia

^6^ Academy of Sciences of Sakha (Yakutia), Yakutsk 677007, Russia

^7^ College of Food and Biology, Jingchu University of Technology, Jingmen, Hubei 448000, China

^8^ Faculty of Veterinary and Agricultural Science, The University of Melbourne, Parkville, Victoria 3010, Australia

^9^ The Davies Research Centre, School of Animal and Veterinary Sciences, University of Adelaide, Roseworthy, SA 5371, Australia

^10^ Institute of Animal Husbandry and Veterinary Science, Tibet Academy of Agricultural and Animal Husbandry Sciences, Lhasa 850000, China

^11^ College of Veterinary Medicine, Northwest A&F University, Yangling 712100, China

^12^ Qinghai Animal and Veterinary Sciences Work Station, Yushu 815000, China

^13^ Key Laboratory of Genetic Evolution & Animal Models and Yunnan Key Laboratory of Molecular Biology of Domestic Animals, Kunming Institute of Zoology, Chinese Academy of Sciences, Kunming 650223, China

^14^ Kunming College of Life Science, University of Chinese Academy of Sciences, Kunming 650204, China

^15^ Sino-Africa Joint Research Center, Chinese Academy of Sciences, Kunming 650204, China

^#^These authors contributed equally: Luyang Sun, Liangliang Jin, and Ting Sun.

*Correspondence: mayun@nxu.edu.cn (Yun Ma); xiaxiaoting@nwafu.edu.cn (Xiaoting Xia); leichuzhao1118@nwafu.edu.cn (Chuzhao Lei); ningbochen@nwafu.edu.cn (Ningbo Chen)

**METHODS**

**Sample preparation and DNA resequencing data**

We collected and extracted 76 DNA samples from indigenous cattle representing 18 breeds/populations. Paired-end libraries were generated for each sample using standard procedures. The average insert size was 500 bp, and the read length was 150 bp. All libraries were sequenced on the Illumina HiSeq X platform to an average raw read sequencing depth of 10×. The sequencing depth ranged from 7.71× to 14.34× (average: 10.20× per genome). The new whole-genome sequences generated in this study for 76 samples have been deposited at the National Center for Biotechnology Information BioProject database (https://www.ncbi.nlm.nih.gov/bioproject) under the BioProject accession numbers PRJNA905718, PRJNA1085861, and PRJNA658727. We combined our new data with those from 668 publicly available whole genomes of 83 breeds/populations. The average sequencing depth was 11.28×, ranging from 7.15× to 33.99×, per genome. The details of the data mentioned above and other downloaded publicly available data used in this study are provided in Table S1-2.

**Variant calling evaluation**

A total of 744 cattle samples were used for variant discovery. We generated genotype data following the 1000 Bull Genomes Project Run 8 guidelines (<http://www.1000bullgenomes.com/>). We removed low-quality bases and artifact sequences via Trimmommatic v0.39 [1], and all the clean reads were mapped to the taurine reference assembly (ARS-UCD1.2) via BWA-MEM v2-2.2.1) [2] with default parameters. We then used SAMtools v1.17 [3] to sort bam files. For the mapped reads, potential PCR duplicates were identified using ‘MarkDuplicates’ of Picard v2.20.2 (<http://broadinstitute.github.io/picard>). The ‘BaseRecalibrator’ and ‘PrintReads’ of Genome Analysis Toolkit (GATK, v4.4.0.0)[4] were used to perform base quality score recalibration (BQSR) with the known variant file (ARS1.2PlusY_BQSR_v3.vcf.gz) provided by the 1000 Bull Genomes Project.

For SNP calling, we created GVCF files using ‘HaplotypeCaller’ in GATK with the ‘-ERC GVCF’ option. We called SNPs from combined GVCF files using ‘GenotypeGVCFs’ and ‘SelectVariants’. The following criteria were applied to all SNPs: (1) mean sequencing depth (for all individuals) > 1/3× and < 3×; (2) variant confidence/quality by depth > 2; (3) RMS mapping quality (MQ) > 40.0; (4) Phred-scaled *P* value using Fisher’s exact test to detect strand bias < 60; (5) Z score from the Wilcoxon rank sum test of Alt vs. Ref read MQs (MQRankSum) > −12.5; and (6) Z score from the Wilcoxon rank sum test of Alt vs. Ref read position bias (ReadPosRankSum) > −8. We then filtered out nonbiallelic SNPs and SNPs with missing genotype rates > 0.1. Imputation and phasing of SNPs were simultaneously performed via BEAGLE v4.0 with default parameters, and SNPs were filtered with DR2 < 0.9 [5]. The remaining high-quality SNPs were annotated according to their positions via SnpEff v4.3 and used in the downstream analysis [6].

**Population genetics**

The VCF file containing 34,902,931 SNPs was converted into PLINK format with VCFtools v0.1.17 [7]. The genome-wide nucleotide diversity of cattle belonging to different geographic groups was estimated with VCFtools with a scanning window of 50 kb and a step size of 20 kb. Genetic distances between breeds/populations were calculated with the *F*_ST_ estimates and inbreeding coefficients were analyzed via PLINK (v1.90b6.21). To construct a phylogenetic tree, we selected 34,902,931 SNPs with MAF > 0.01. We constructed a phylogenetic tree using the NJ method with a matrix of pairwise genetic distances and visualized it with MEGA7 [8]. For principal component analysis (PCA) and admixture analysis, we performed LD-based pruning for the genotype data using the --indep-pairwise 50 10 0.1 option of PLINK. PCA was performed on the same SNP dataset (7,057,330 SNPs) using the smartpca program in EIGENSFOT v5.052 [9]. The population structure was investigated using ADMIXTURE v1.3.0 [10]. To determine of the most likely cluster (K) number, the cross-validation procedure in ADMIXTURE was run for values of K from 2 to 10. The nucleotide diversity of the five screened breeds was calculated. A total of 731 cattle samples with 32,506,509 SNPs were used to calculate the genetic distance between breeds/populations via *F*_ST_ estimates. An unrooted network based on the distance matrix was analyzed via the neighbor net algorithm SplitsTree4 [11].

**Collection of environment factors**

Environmental variables were obtained for each breed according to the coordinates of the sampling locations. We obtained a total of 28 environmental variables related to temperature (6), humidity (6), precipitation (4), wind speed (3), air pressure (1), radiation and cloud cover (2), evapotranspiration (2), soil and surface (3), and vegetation (1) (Table S5), representing the extremes and seasonality of temperature and precipitation, relative air humidity, water vapor pressure, pressure, and aridity. Seven datasets of environmental variables were downloaded from CRU (<https://www.uea.ac.uk/groups-and-centres/climatic-research-unit>), and 21 environmental variables were downloaded from NOAA PSL (<https://psl.noaa.gov/>). The resolution of the environmental variables ranged from 0.5° to 2.5° (grids). This study used netCDF4 to extract monthly climate data from .nc files over the past 40 years. During data extraction, invalid values (such as missing data) were excluded. Then, on the basis of the extracted monthly data, we calculated the average climate value for each year, and further counted the maximum and minimum values in December each year, as well as the difference between the highest and lowest values each year, to fully assess the trend and magnitude of climate change. Kernel density plots of the environmental variables were used to display the distribution of the climate supporting cattle survival (Figure S5-S9). To study the correlations among the environmental variables, we use RStudio [12] to conduct Spearman rank correlation test on the annual mean value of environmental variables in the dataset to evaluate the long-term linear relationship between the variables. The ggcorrplot, corrplot and viridis packages were used to visualize the correlation matrix. The gord package was then used for PCA of all data, including annual mean, maximum, minimum, and their differences.

**Genome-wide environmental association study**

We performed final screening of 731 cattle samples using PLINK. According to the calculated heterozygosity, the individuals with high heterozygosity were filtered, and the heterozygosity distribution was calculated. Large deviations may indicate contamination of the sample and inbreeding. There was no deviation from ± 3 SD in the mean heterozygosity of our samples. Moreover, a value of pihat > 0.2 was calculated via PLINK, which involves an IBS/IBD computation to count approximately 1 cattle genome that may have parent‒child relationships and remove them. We ended up with 32,300,781 high-quality SNPs for subsequent analysis.

The annual average, minimum, maximum and differences between the maximum and minimum CVs of each variety were calculated as parameters (Table S6). We used the LMM [13] , LFMM [14] BayPass [15] methods to identify markers that respond to climate adaptation while controlling for confounding factors due to global genetic differentiation. These complementary methodologies offer distinct advantages: traditional GWAS and LFMM methods can incorporate PC analysis or ancestry judgments as covariates to reduce false positives caused by the population structure, while BayPass software package can overcome some of the limitations of GEA analysis and can also consider the correlation between allele frequency and environmental variables.

The primary association analysis was carried out via the GEMMA software package [13]. The mixed linear model assumes the following model: *y* = *Wα* + *xβ* + *u* + *ε*; u ∼ MVN*_n_*(0, λτ ^−1^*K*), *ε* ∼ MVN*_n_* (0, τ ^−1^ I*_n_*)*.* where *y* is a vector of phenotypes for *n* individuals; W = (w1, · · · , wc) is an *n*×*c* matrix of covariates (fixed effects) including a column of 1 s; *α* is a *c*-vector of fixed effects representing marker effects; *x* is an *n*-vector of marker genotypes; *β* is the effect size of the marker; *u* is an n-vector of random effects; *ε* is an n-vector of errors; τ^−1^ is the variance of the residual errors; λ is the ratio between the two variance components; *K* is a known n × n relatedness matrix; and I_n_ is an n × n identity matrix. MVN_n_ denotes the n-dimensional multivariate normal distribution. To account for population stratification and potential confounding, the top 20 principal components (PCs) were included as covariates in the GEMMA model. This number of PCs was selected as it captured 99.91% of the total genetic variance in our dataset, sufficient to model the major axes of population structure. PCA was chosen for this purpose due to its computational efficiency for large-scale genomic data and its established role as a standard method in population genomics. The top twenty PCs and feeding regimes were defined as the *S* matrix. The standardized genetic relationship matrix was calculated as the *K* matrix. The alternative hypothesis was H_1_ : β ≠ 0 against the null hypothesis H_0_ : β = 0 for each SNP in turn using Wald, one of the three commonly used test statistics. GEMMA obtains either the restricted maximum likelihood estimate (REML) of λ and β, and outputs the corresponding p value. We also calculated the correlations between SNPs and CVs using algorithms implemented in the computer program LFMM [14].We applied the LFMM algorithm and calculated the |z| scores for all of the SNPs using 100 sweeps for burn-in and 1,000 additional sweeps. The optimal latent factor number (K) was determined by performing (PCA of the genotype data. Specifically, we conducted PCA using the prcomp function in R and plotted the variance explained by each principal component (PC). The optimal K value (K = 6), indicating the number of significant latent factors in the data, was identified as the point where the variance explained by the PCs began to plateau. To maintain a stringent control for genome-wide multiple testing across all methods, a Bonferroni correction was applied. The genome-wide significance threshold for both the GEMMA (LMM) and LFMM analyses was set at α = 0.05 divided by the total number of SNPs tested (0.05/*N* _total_). SNPs with a *p* value below this threshold were considered statistically significant.

In BayPass 2.41[15], each autosome was partitioned into multiple subdatasets, with no subdataset containing more than 100,000 SNPs, resulting in a total of 377 subdatasets for the entire dataset. BayPass was executed under the core model, with three independent runs performed for each chromosomal dataset (with the -seed option) (Figure S12A). The association between individual SNPs and climatic adaptation were subsequently assessed using the auxiliary covariate (AUX) model, which incorporates the overall covariance matrix Ω computed under the core model. To ensure consistency, the Forstner and Moonen distance (FMD)[16] was employed to evaluate the results of the three independent runs, utilizing the R function fmd.dist() to measure the distance between pairs of covariance matrices (Ω). The covariance matrices from three distinct seed runs across the 377 subdatasets were compared, revealing consistently low FMD values for all comparisons (Figure S12B). The median Bayes factor (BF), expressed in decibels (dB = 10log10BF), was calculated as the median of the three independent BayPass runs, with values exceeding 20 considered as "decisive" evidence for association generally [15], and we set a more stringent threshold of 30 dB for SNPs to limit the number of false positives.

To ensure these SNPs were associated with environmental adaptation rather than strong population differentiation, we applied a stringent filtering criterion based on the XtX statistic from the BayPass core model. We excluded SNPs with XtX values above the FDR = 0.5% threshold (XtX > 151) (Figure S12), as these loci are likely to reflect selection or genetic drift rather than environmental adaptation. By integrating the results from the three methods (LMM, LFMM, and BayPass), we identified a total of 3165 high-confidence SNPs (LMM/LFMM: *p* < 1.55E^-9^; BayPass: BF > 30) supported by at least two methods (Table S7).

**Identification of causal variation in environmental adaptation in domestic cattle**

A total of 32 published ATAC-seq datasets were obtained from NCBI, encompassing eight different tissues (adipose, liver, spleen, lung, muscle, cerebral cortex, hypothalamus and thyroid gland) from two adult Hereford cattle, three postpuberty Brahman heifer cattle, three adult Weizhou cattle (Unpublished) and two adult Luxi cattle (Table S9-10). After the adapters were checked and trimmed with Trim Galore (v.0.6.10) (https://github.com/FelixKrueger/TrimGalore), the ATAC-seq clean reads were mapped to ARS-UCD1.2 using Bowtie2 (v.2.4.5) [17]. BAM files were sorted using SAMtools (v.1.3) [3], and duplicate alignments were removed with Picard (v2.20.2) (http://broadinstitute.github.io/picard). We performed RPKM normalization using deepTools for both ATAC-seq BAM files. The peaks for individual replicates were called separately with MACS2 (v2.2.7.1) [18] and then merged across three duplicates within the same tissue using bedtools. A total of 61 published ChIP-seq datasets were obtained from the same samples of two adult Hereford cattle (Table S9-10). The same pipeline was used for processing the ChIP-seq data. The open chromatin region peaks were observed via the Integrative Genomics Viewer (IGV) [19]. To compare gene expression between cold-adapted and heat-adapted cattle, we integrated two publicly available Hereford cattle muscle tissue RNA-Seq datasets with three newly sequenced Weizhou cattle muscle tissue datasets (Table S9-10). Illumina HiSeq 2500 System sequencing was used to sequence the total RNA from Weizhou cattle samples. The raw reads were processed to obtain clean reads with FASTP v0.12.3[20]. Based on clean and high-quality data, HISAT2 v2.0.3-beta [21] and StringTie v1.2.2 [22] were used to compare the sequences with the reference genome (ARS-UCD1.2). Transcript-level counts were normalized to Transcripts Per Million (TPM) values using gene lengths obtained from the genome annotation. Differential gene expression analysis was performed using the limma v3.50.3 package [23]. The empirical Bayes moderated t-test was used to evaluate the gene expression differences between the two cattle groups. *P* values were adjusted for multiple testing using the Benjamini-Hochberg (BH) method to control the false discovery rate (FDR).

To detect common causal variations in gene expression and environmentally adaptive complex traits, we used *cis*-eQTL data [24] from <https://cgtex.roslin.ed.ac.uk/>. To identify SNPs, we compared all significant *cis*-eQTLs in the mammary, spleen, kidney, muscle indicus, muscle taurus, hypothalamus, pituitary, muscle cross, muscle, uterus, macrophage, adipose, jejunum, oviduct, leukocyte, rumen, ovary, intramuscular fat, testis, embryo, blood, liver, lung, milk cell, monocyte, lymph node and mammary L tissues using bedtools intersect-v (Table S11). We further conducted colocalization analysis of eQTLs and climate-associated SNPs using Coloc [25] with the function coloc.abf. We obtained posterior probability values for the H4 case (PP.H4), i.e., both traits (GWAS traits and eQTLs) are associated and share a single causal variant. We kept both traits with PP.H4 > 0.8, which can be considered shared causal variation.

**Screening for Candidate Genes Associated with Environmental Adaptations**

We identified important SNPs within genes using bedtools intersect and SnpEff [6]. To determine whether the identified candidate genes are associated with specific functional categories, we used Kyoto Encyclopedia of Genes and Genomes (KEGG) and Gene Ontology (GO) in the DAVID program [26] to conduct functional enrichment analyses. Functional categories with a BH false discovery rate (FDR) adjusted *p* value of less than 0.1 were considered significantly enriched.

To identify population-specific adaptive variants, we applied stringent filtering criteria based on derived allele frequencies (DAF), tailored to the genetic structure of each focal population. For Yakutian cattle and N’Dama cattle, SNPs were selected if they had a DAF > 0.7 in the target population and DAF < 0.3 in all other cattle populations. Given the genetic heterogeneity within Tibetan cattle—which includes both *Bos taurus* and *Bos indicus* ancestry—we adopted a modified approach. SNPs were considered Tibetan-specific if they had a DAF < 0.3 in all non-Tibetan populations, and DAF > 0.5 in at least two Tibetan cattle breeds.

To evaluate selection at the BTA18 candidate region, LD decay was analyzed using PopLDdecay v3.42 [27]. We calculated pairwise r² values within a 1 Mb window (14.0–15.0 Mb) that includes the identified sweep region. As a control, several 1 Mb regions were randomly selected across the genome. LD decay curves were generated separately for cold- and heat-adapted populations.

Furthermore, we calculated nucleotide diversity (π) and Tajima's D statistics for the candidate regions across different populations using a small sliding window to integrate the top signals, verifying that the target genomic regions in the focal population were more frequently under selection than the corresponding regions in other populations were. We performed extended haplotype homozygosity (EHH) analysis using the software package rehh v3.2.2[28] implemented in R [29]. EHH was computed for the candidate SNPs to evaluate the decay of haplotype homozygosity away from the core allele. To visualize the local linkage disequilibrium (LD) structure of candidate genomic regions, we used LDBlockShow v1.40[30] to generate LD block plots.

**Supplementary Figures**

**Figure S1 The genetic diversity of the Old World of cattle.** (A) Genome-wide nucleotide diversity in different cattle phylogeographic groups obtained using VCFtools. The horizontal line inside the box corresponds to the median of the distribution. (B) Genome-wide inbreeding coefficients in different cattle phylogeographic groups obtained using PLINK. The ten groups are as follows: African indicine (AFI), African taurine (AFT), European taurine (EUT), East Asian taurine (EAT), Tibetan cattle (TB), Eurasian taurine (EUAT), South Asian indicine (SAI), Southeast Asian indicine (SEAI), Central China (CC), and East Asian indicine (EAI).

**Figure S2 Pairwise genetic differentiation (*F*_ST_) among cattle breeds/populations.** Heatmap displays mean pairwise *F*_ST_ values calculated between all cattle breeds/populations represented by more than one individual.

**Figure S3** **Population structure inferred from ADMIXTURE analysis.** The analysis was performed using 7,057,330 LD-pruned SNPs for K = 2 to 10 on 744 individuals from 85 breeds/populations. The ten groups are as follows: African indicine (AFI), African taurine (AFT), European taurine (EUT), East Asian taurine (EAT), Tibetan cattle (TB), Eurasian taurine (EUAT), South Asian indicine (SAI), Southeast Asian indicine (SEAI), Central China (CC), and East Asian indicine (EAI). Breeds are arranged from left to right as: (1) Nari Master, (2) Achai, (3) Bangladesh, (4) Bhagnari, (5) Brahman, (6) Cholistani, (7) Dajal, (8) Dhanni, (9) Gabrialli, (10) Nepal, (11) Red Sindhi, (12) Sahiwal, (13) Tharparkar, (14) Thawalam, (15) Burma, (16) Dehong, (17) Dianzhong, (18) Jiangcheng, (19) Jinghong, (20) Dabieshan, (21) Guangfeng, (22) Jian, (23) Longlin, (24) Minnan, (25) Nandan, (26) Tiantai, (27) Wannan, (28) Weizhou, (29) Wenlinggaofeng, (30) Wenshan, (31) Xiangxi, (32) Yiling, (33) Leiqiong, (34) Abergelle, (35) Arado, (36) Arsi, (37) Begait, (38) Boran, (39) Choke, (40) Erob, (41) Ethiopia, (42) Goffa, (43) Kenana, (44) Ogaden, (45) Raya, (46) Semien, (47) Muturu, (48) N’Dama, (49) Mishima, (50) Hanwoo, (51) Yakutian, (52) Yanbian, (53) Anxi, (54) Chaidamu, (55) Kazakh, (56) (Inner) Mongolian, (57) Mongolian, (58) Jersey, (59) Angus, (60) Eastern Finncattle, (61) Gelbvieh, (62) Hereford, (63) Holstein, (64) Red Angus, (65) Simmental, (66) Western Finncattle, (67) Changdu, (68) Dingjie, (69) Diqing, (70) Yushu, (71) Shigatse, (72) Bashan, (73) Bohai black, (74) Dengchuan, (75) Huangpi, (76) Jiaxian red, (77) Lingnan, (78) Luxi, (79) Nanyang, (80) Qinchuan, (81) Sanjiang, (82) Weining, (83) Zaobei, (84) Zaosheng, (85) Zhaotong.

 **Figure S4** **Population genetic results.** (A) Neighbor-joining tree constructed using whole-genomic autosomal SNP data. (B) Genome-wide nucleotide diversity in five breeds of cattle before and after the removal of hybridized individuals using VCFtools. The results obtained after the hybrid individuals were removed are presented in brown font. The horizontal line inside the box corresponds to the median of the distribution, and the upper and lower parts of the box are the first and third quartiles, respectively. Data points outside the whiskers can be considered outliers.

**Figure S5 *F*_ST_ among different cattle breeds/groups after individual selection.** Mean pairwise *F*_ST_ values between cattle breeds/populations represented by more than one animal of 731 cattle samples with 32,506,509 SNPs.

**Figure S6 Monthly density distributions of climate variables: STR, OLR, POTTMP, TMN, TMAX, and TMIN.** Kernel density plots of six temperature-related environmental variables for each month: Surface Temperature (STR), Outgoing Longwave Radiation (OLR), Potential Temperature (POTTMP), Air Temperature (TMN), Maximum Temperature (TMAX), and Minimum Temperature (TMIN).

**Figure S7 Monthly density distributions of climate variables: PDSI, QMAX, SHUM, QMIN, RHUM, and PRWTR.** Kernel density plots of six moisture-related environmental variables for each month: Palmer Drought Severity Index (PDSI), Maximum Specific Humidity (QMAX), Specific Humidity (SHUM), Minimum Specific Humidity (QMIN), Relative Humidity (RHUM), and Precipitable Water (PRWTR).

**Figure S8 Monthly density distributions of climate variables: PRATE, PEVPR, WSPD, UWND, SRFP, and VAP.** Kernel density plots of six additional environmental variables for each month: Precipitation Rate (PRATE), Potential Evaporation Rate (PEVPR), Wind Speed (WSPD), U-Wind Component (UWND), Surface Pressure (SRFP), and Vapor Pressure (VAP).

**Figure S9 Monthly density distributions of climate variables: PET, FRS, SOILW, VWND, DTR, and WET.** Kernel density plots of six additional environmental variables for each month: Potential Evapotranspiration (PET), Ground Frost Frequency (FRS), Soil Moisture (SOILW), V-Wind Component (VWND), Diurnal Temperature Range (DTR), and Wet Day Frequency (WET).

**Figure S10 Monthly density distributions of climate variables: CLD, SNOW, PRE, and VEG.** Kernel density plots of four additional environmental variables for each month: Cloud Cover (CLD), Snow Cover (SNOW), Total Precipitation (PRE), and Vegetation Cover (VEG).

**Figure S11 Principal component analysis of environmental variables across cattle breeds/populations.** (A) PCA of 28 environmental variables for 85 cattle breeds/populations, illustrated by PC1 against PC2. The lower right corner is the eigenvector corresponding to the original climate data. (B) Eigenvalues corresponding to principal component coordinates.

**Figure S12 Results of the core model from BayPass analysis.** (A) Manhattan plot of XtX statistics, showing the median values from three independent runs, used to detect signals of natural selection. (B) Consistency of covariance matrices (Ω) across three independent BayPass runs assessed by Forstner and Moonen distance (FMD). Boxplots show FMD values from 377 sub-datasets using three different random seeds, indicating high concordance among runs.

**Figure S13 Part of the results from the genome-wide environmental association analysis.** (A) Distribution of candidate SNP-environment associations from BayPass analysis. Boxplot shows the Bayes factor (BF) distribution of candidate SNPs across environmental variables, with the estimated false positive rate (FPR) indicated. (B) Functional classification of candidate SNPs. (C) Gene Ontology and KEGG pathway enrichment analysis of candidate genes.

**Figure S14 Heatmap of lineage-specific allele frequencies for environmental adaptation SNPs in Yakutian and Tibetan cattle.** The heatmap displays the allele frequencies of all candidate environmental adaptation SNPs identified as lineage-specific in Yakutian and Tibetan cattle. Rows represent individual SNP loci, and columns represent cattle breeds/populations. Breeds are grouped at the top by their major lineage classifications. The breed names for Yakutian and Tibetan cattle are highlighted in red for emphasis.

**Figure S15 Analysis of selection signals and linkage disequilibrium decay in temperature-adapted cattle groups.** (A) Selective signals of cattle groups based on Tajima’s D values and nucleotide diversity (θπ). The taurine cattle groups included EAT, EUAT, and EUT. The indicine cattle groups included SAI, SEAI, EAI, and AFI. The gene annotation information within the scanning area is displayed at the bottom, and the genes where the significant sites are located are highlighted in red. (B) Comparison of linkage disequilibrium (LD) decay in the 14.0–15.0 Mb region of BTA18 and randomly selected genomic regions across different temperature-adapted cattle groups. Cold-adapted populations exhibit slower LD decay in the candidate region, suggesting possible selection signals.

 **Figure S16 Heatmap of lineage-specific allele frequencies for environmental adaptation SNPs in N'Dama cattle.** The heatmap illustrates the allele frequencies of candidate environmental adaptation SNPs identified as lineage-specific in N'Dama cattle. Breeds are grouped at the top by their major lineage classifications. Only the N'Dama breed label is explicitly annotated on the x-axis for clarity.

**Figure S17 Extended haplotype homozygosity (EHH) decay and haplotype bifurcation around rs111440168.** Decay of extended haplotype homozygosity (EHH) per site around the candidate variant rs111440168 in N'Dama cattle compared to other taurine cattle. Bifurcation diagram of core haplotypes derived from the same genomic region, illustrating the haplotype structure and differentiation between N'Dama and other taurine populations.

**Figure S18 Significant genomic region association with temperature of *SLAMF1* and *CD84* gene.** (A) The Manhattan and QQ plots showing the associations of 83 cattle breeds with PRATE climate variable. (B) Haplotype structures for significant genomic region in cattle groups. (C) Allele frequency geographical distribution of the missense mutations rs110202396 and rs10897540.

**Figure S19 Allele frequency geographical distribution of the intron mutations rs210877007, rs382149931 and rs110707290 on the *ARFGEF3* gene.** The colors on the map represent the 40-year average potential evapotranspiration degree of different regions in June. The reference site is dark green and the mutation site is orange-red.

**Figure S20 Functional and population genetics analysis of the FARP2 gene locus on BTA3.** (A) eQTLs of the *FARP2* gene in the pituitary gland and GWAS loci of the PDSI CV in cattle on BTA3. (B)Allele frequency geographical distributions of rs476116768 in *FARP2* with global cattle breeds/populations.

**Figure S21 Tissue‑specific chromatin profiles at the *FARP2* insulator variant rs476116768 visualized.** Integrated Genomics Viewer (IGV) tracks show ChIP‑seq signals across four bovine tissues from Hereford cattle, centered on the insulator region containing variant rs476116768 in the *FARP2* gene. Numbers in brackets indicate normalized ChIP‑seq peak intensities.

**REFERENCES**

1. Bolger, Anthony M, Marc Lohse, Bjoern Usadel. 2014. “Trimmomatic: a flexible trimmer for Illumina sequence data.” *Bioinformatics* 30: 2114-2120. <https://doi.org/10.1093/bioinformatics/btu170>

2. Li, Heng, Richard Durbin. 2009. “Fast and accurate short read alignment with Burrows-Wheeler transform.” *Bioinformatics* 25: 1754-1760. <https://doi.org/10.1093/bioinformatics/btp324>

3. Li, Heng, Bob Handsaker, Alec Wysoker, Tim Fennell, Jue Ruan, Nils Homer, Gabor Marth, Goncalo Abecasis, Richard Durbin, 1000 Genome Project Data Processing Subgroup. 2009. “The Sequence Alignment/Map format and SAMtools.” *Bioinformatics* 25: 2078-2079. <https://doi.org/10.1093/bioinformatics/btp352>

4. McKenna, Aaron, Matthew Hanna, Eric Banks, Andrey Sivachenko, Kristian Cibulskis, Andrew Kernytsky, Kiran Garimella, et al. 2010. “The Genome Analysis Toolkit: a MapReduce framework for analyzing next-generation DNA sequencing data.” *Genome research* 20: 1297-1303. <https://doi.org/10.1101/gr.107524.110>

5. Browning, Sharon R, Brian L Browning. 2007. “Rapid and accurate haplotype phasing and missing-data inference for whole-genome association studies by use of localized haplotype clustering.” *American journal of human genetics* 81: 1084-1097. <https://doi.org/10.1086/521987>

6. Cingolani, Pablo, Adrian Platts, Le Lily Wang, Melissa Coon, Tung Nguyen, Luan Wang, Susan J Land, Xiangyi Lu, Douglas M Ruden. 2012. “A program for annotating and predicting the effects of single nucleotide polymorphisms, SnpEff: SNPs in the genome of Drosophila melanogaster strain w1118; iso-2; iso-3.” *Fly (Austin)* 6: 80-92. <https://doi.org/10.4161/fly.19695>

7. Danecek, Petr, Adam Auton, Goncalo Abecasis, Cornelis A Albers, Mark A DePristo Eric Banks, Robert E Handsaker, Gerton Lunter, et al. 2011. “The variant call format and VCFtools.” *Bioinformatics* 27: 2156-2158. <https://doi.org/10.1093/bioinformatics/btr330>

8. Kumar, Sudhir, Glen Stecher, Koichiro Tamura. 2016. “MEGA7: Molecular Evolutionary Genetics Analysis Version 7.0 for Bigger Datasets.” *Molecular biology and evolution* 33: 1870-1874. <https://doi.org/10.1093/molbev/msw054>

9. Price, Alkes L, Nick J Patterson, Robert M Plenge, Michael E Weinblatt, Nancy A Shadick, David Reich. 2006. “Principal components analysis corrects for stratification in genome-wide association studies.” *Nature genetics* 38: 904-909. <https://doi.org/10.1038/ng1847>

10. Alexander, David H, Kenneth Lange. 2011. “Enhancements to the ADMIXTURE algorithm for individual ancestry estimation.” *BMC Bioinformatics* 12: 246. <https://doi.org/10.1186/1471-2105-12-246>

11. Al-Mamun, Hawlader A, Paul Kwan, Samuel A Clark, Mohammad H Ferdosi, Ross Tellam, Cedric Gondro. 2015. “Genome-wide association study of body weight in Australian Merino sheep reveals an orthologous region on OAR6 to human and bovine genomic regions affecting height and weight.” *Genetics selection evolution* 47: 66. <https://doi.org/10.1186/s12711-015-0142-4>

12. Loraine, Ann E, Ivory Clabaugh Blakley, Sridharan Jagadeesan, Jeff Harper, Gad Miller, Nurit Firon. 2015. “Analysis and visualization of RNA-Seq expression data using RStudio, Bioconductor, and Integrated Genome Browser.” *Methods in molecular biology* 1284: 481-501. <https://doi.org/10.1007/978-1-4939-2444-8_24>

13. Zhou, Xiang, Matthew Stephens. 2012. “Genome-wide efficient mixed-model analysis for association studies.” *Nature genetics* 44: 821-824. <https://doi.org/10.1038/ng.2310>

14. Frichot, Eric, Sean D Schoville, Guillaume Bouchard, Olivier François. 2013. “Testing for associations between loci and environmental gradients using latent factor mixed models.” *Molecular biology and evolution* 30: 1687-1699. <https://doi.org/10.1093/molbev/mst063>

15. Gautier, Mathieu. 2015. “Genome-Wide Scan for Adaptive Divergence and Association with Population-Specific Covariates.” *Genetics* 201: 1555-1579. <https://doi.org/10.1534/genetics.115.181453>

16. Förstner, Wolfgang, Boudewijn Moonen. 2003. A Metric for Covariance Matrices. *Geodesy-The Challenge of the 3rd Millennium* Springer Berlin Heidelberg, 299-309. <https://doi.org/10.1007/978-3-662-05296-9_31>

17. Langmead, Ben, Steven L Salzberg. 2012. “Fast gapped-read alignment with Bowtie 2.” *Nature methods* 9: 357-359. <https://doi.org/10.1038/nmeth.1923>

18. Zhang, Yong, Tao Liu, Clifford A Meyer, Jérôme Eeckhoute, David S Johnson, Bradley E Bernstein, Chad Nusbaum, et al. 2008. “Model-based analysis of ChIP-Seq (MACS).” *Genome biology* 9: R137. <https://doi.org/10.1186/gb-2008-9-9-r137>

19. Thorvaldsdóttir, Helga, James T Robinson, Jill P Mesirov. 2013. “Integrative Genomics Viewer (IGV): high-performance genomics data visualization and exploration.” *Brief Bioinform* 14: 178-192. <https://doi.org/10.1093/bib/bbs017>

20. Chen, Shifu. 2023. “Ultrafast one-pass FASTQ data preprocessing, quality control, and deduplication using fastp.” *iMeta* 2: e107. <https://doi.org/10.1002/imt2.107>

21. Kim, Daehwan, Ben Langmead, Steven L Salzberg. 2015. “HISAT: a fast spliced aligner with low memory requirements.” *Nature methods* 12: 357-360. <https://doi.org/10.1038/nmeth.3317>

22. Pertea, Mihaela, Geo M Pertea, Corina M Antonescu, Tsung-Cheng Chang, Joshua T Mendell, Steven L Salzberg. 2015. “StringTie enables improved reconstruction of a transcriptome from RNA-seq reads.” *Nature biotechnology* 33: 290-295. <https://doi.org/10.1038/nbt.3122>

23. Ritchie, Matthew E, Belinda Phipson, Di Wu, Yifang Hu, Charity W Law, Wei Shi, Gordon K Smyth. 2015. “limma powers differential expression analyses for RNA-sequencing and microarray studies.” *Nucleic Acids Research* 43: e47. <https://doi.org/10.1093/nar/gkv007>

24. Liu, Shuli, Yahui Gao, Oriol Canela-Xandri, Sheng Wang, Ying Yu, Wentao Cai, Bingjie Li, et al. 2022. “A multi-tissue atlas of regulatory variants in cattle.” *Nature genetics* 54: 1438-1447. <https://doi.org/10.1038/s41588-022-01153-5>

25. Giambartolomei, Claudia, Damjan Vukcevic, Eric E Schadt, Lude Franke, Aroon D Hingorani, Chris Wallace, Vincent Plagnol. 2014. “Bayesian test for colocalisation between pairs of genetic association studies using summary statistics.” *PLoS genetics* 10: e1004383. <https://doi.org/10.1371/journal.pgen.1004383>

26. Huang, Da Wei, Brad T Sherman, Richard A Lempicki. 2009. “Systematic and integrative analysis of large gene lists using DAVID bioinformatics resources.” *Nature protocols* 4: 44-57. <https://doi.org/10.1038/nprot.2008.211>

27. Zhang, Chi, Shan-Shan Dong, Jun-Yang Xu, Wei-Ming He, Tie-Lin Yang. 2019. “PopLDdecay: a fast and effective tool for linkage disequilibrium decay analysis based on variant call format files.” *Bioinformatics* 35: 1786-1788. <https://doi.org/10.1093/bioinformatics/bty875>

28. Sabeti, Pardis C., David E. Reich, John M. Higgins, Haninah Z. P. Levine, Daniel J. Richter, Stephen F. Schaffner, Stacey B. Gabriel, et al. 2002. “Detecting recent positive selection in the human genome from haplotype structure.” *Nature* 419: 832-837. <https://doi.org/10.1038/nature01140>

29. Gautier, Mathieu, Renaud Vitalis. 2012. “rehh: an R package to detect footprints of selection in genome-wide SNP data from haplotype structure.” *Bioinformatics* 28: 1176-1177. <https://doi.org/10.1093/bioinformatics/bts115>

30. Dong, Shan-Shan, Wei-Ming He, Jing-Jing Ji, Chi Zhang, Yan Guo, Tie-Lin Yang. 2021. “LDBlockShow: a fast and convenient tool for visualizing linkage disequilibrium and haplotype blocks based on variant call format files.” *Brief Bioinform* 22: <https://doi.org/10.1093/bib/bbaa227>
